# Influence of Ovarian Hormones on Cortical Spreading Depression and Its Suppression by L-kynurenine in Rat

**DOI:** 10.1371/journal.pone.0082279

**Published:** 2013-12-10

**Authors:** Virginie Chauvel, Jean Schoenen, Sylvie Multon

**Affiliations:** 1 Cephalic Pain Unit of GIGA-Neurosciences, Liège University, Liège, Belgium; 2 Headache Research Unit, Dept. of Neurology, Liège University, CHR Citadelle, Liège, Belgium; Inserm U837, France

## Abstract

Migraine is sexually dimorphic and associated in 20–30% of patients with an aura most likely caused by cortical spreading depression (CSD). We have previously shown that systemic L-kynurenine (L-KYN), the precursor of kynurenic acid, suppresses CSD and that this effect depends on the stage of the estrous cycle in female rats. The objectives here are to determine the influence of ovarian hormones on KCl-induced CSD and its suppression after L-KYN by directly modulating estradiol or progesterone levels in ovariectomized rats. Adult female rats were ovariectomized and subcutaneously implanted with silastic capsules filled with progesterone or 17β-estradiol mixed with cholesterol, with cholesterol only or left empty. Two weeks after the ovariectomy/capsule implantation, the animals received an i.p. injection of L-KYN (300 mg/kg) or NaCl as control. Thirty minutes later CSDs were elicited by applying KCl over the occipital cortex and recorded by DC electrocorticogram for 1 hour. The results show that both estradiol and progesterone increase CSD frequency after ovariectomy. The suppressive effect of L-KYN on CSD frequency, previously reported in normal cycling females, is not found anymore after ovariectomy, but reappears after progesterone replacement therapy. Taken together, these results emphasize the complex role of sex hormones on cortical excitability. The CSD increase by estradiol and, more surprisingly, progesterone may explain why clinically migraine with aura appears or worsens during pregnancy or with combined hormonal treatments.

## Introduction

Migraine is the most common neurological disorder and occurs in about 15% of the population with a female/male ratio of 3/1 [1]. The mechanisms of sexual dimorphism in migraine are not well understood, but ovarian hormones, especially estrogens [2], seem to play a key role. Indeed, migraine in women is influenced by menarche, menstruation, pregnancy, menopause, oral contraceptive use, and hormonal replacement therapy [3]. Sex steroids, however, may differentially modulate migraine with aura (MA) and without aura (MO). In contrast to MO, MA is favored by hyperestrogenic states: it can appear during pregnancy [4] and is worsened by oral contraceptives [5].

Strong evidence from clinical correlations with functional brain imaging studies suggests that the migraine aura is due to cortical spreading depression (CSD) originating in the occipital cortex [6]. CSD is a slowly progressing wave (3–5 mm/min) of neurono-glial depolarization followed by a long-lasting suppression of neuronal activity and excitability [7]. It has been shown that gonadal steroids can modulate CSD susceptibility. In female mice CSD thresholds are lower than in males [8]. Estrogens are considered responsible for the higher CSD propagation velocity in Wistar audiogenic rats [9]. The increased susceptibility to CSD of female FHM1 knock-in mice is abolished by ovariectomy whereafter it is partially restored by estradiol treatment [10].

Glutamate and glutamate receptors play a pivotal role in the initiation and propagation of CSD. Glutamate and N-Methyl-D-aspartate (NMDA) trigger CSD, while NMDA receptor antagonists inhibit CSD initiation and propagation [11]. We showed previously that CSD is suppressed by systemic administration of L-kynurenine (L-KYN) [12]. L-kynurenine is the precursor of kynurenic acid that is an endogenous NMDA receptor antagonist [13]. As kynurenic acid penetrates poorly the blood–brain barrier [14], L-KYN is given systemically to rats to increase the brain concentrations of kynurenic acid [15]. In our study, the CSD-suppressive effect of L-KYN was more pronounced in females than in males and in females it differed between the phases of the estrous cycle [12].

In the present study, we have therefore investigated separately the modulating effect of estrogen and progesterone on CSD frequency in ovariectomized rats, as well as the influence of these hormones on the CSD suppression by L-KYN.

## Materials and Methods

### Animals

A total of 66 female adult Sprague-Dawley rats were used in this study. They were raised and maintained under standard laboratory conditions, with tap water and regular rat chow available *ad libitium* on a 12-h dark 12-h light cycle. All animal procedures and care complied with the guidelines of the International Association for the Study of Pain and the European Communities Council (86/609/ECC) and were approved by the Ethics Committee of the Faculty of Medicine of Liège University (ethic protocol number 1233). All surgery was performed under isoflurane or chloral hydrate anesthesia, and all efforts were made to minimize suffering.

### Ovariectomy and hormonal treatments

Animals were bilaterally ovariectomized under isoflurane inhalation (2 to 3% in a flow of 1 l/min of oxygen, Forene®, Abbott, Queenborough, Kent, England) and subcutaneously implanted with silastic capsules. Estrogen implants were 1 cm in length and filled with a 20% 17β-estradiol-cholesterol mixture (E2; Sigma-Aldrich, Steinheim, Germany; n = 18), whereas estrogen control implants were 100% cholesterol (E2Cont; Sigma-Aldrich, Steinheim, Germany, n = 16). Progesterone implants were 3 cm in length and filled with 100% progesterone (P4; Sigma-Aldrich, Steinheim, Germany, n = 16); empty capsules were used as controls (P4Cont, n = 15). Implants of such lengths and concentrations are known to reproduce the physiological peak of proestrus blood levels of hormones [16].

### Experimental protocol

Two weeks after ovariectomy and capsule implantation, each of the before described treatment groups were divided in a subgroup receiving an intraperitoneal (i.p.) injection of 300 mg/kg of L-kynurenine sulphate (“L-KYN”, Sigma, Steinheim, Germany; E2 L-KYN group n = 9; E2Cont L-KYN n = 8; P4 L-KYN n = 8; P4Cont L-KYN n = 7) and another receiving physiological saline (“NaCl”; E2 NaCl group n = 9; E2Cont NaCl n = 8; P4 NaCl n = 8; P4Cont NaCl n = 8) injections as controls. The L-KYN dose was chosen based on previous studies of cortical excitability [12], [17]. We started to elicit and record CSDs 30 minutes after the *i.p.* injections.

CSDs were studied according to the method previously described [18]. Briefly, anesthetized rats (chloral hydrate, 400 mg/kg) were placed in a stereotactic frame (David Kopf Instruments, USA). Rectal temperature was maintained between 36.5 and 37.0°C using a thermostatically controlled heating blanket (ATC 1000®, WPI Inc., USA), heart rate and blood oxygen level were monitor with a rodent oximeter (Kent Scientific corporation, USA). Three 1–2 mm wide burr holes were drilled 2 mm off the midline: 7 mm posterior to bregma (P-7; occipital cortex; stimulation site), 4 mm posterior to bregma (P-4; occipito-parietal recording site) and 1 mm anterior to bregma (A+1; frontal recording site) [19].

We induced CSDs by placing a cotton ball soaked with 1 M KCl over the pial surface at the stimulation site. Cortical direct current (DC) potential shifts and the electrocorticogram were recorded with Ag/AgCl electrodes. The electrical signals were amplified with an ISODAM-8A bioamplifier at a DC-10 kHz band width (WPI Inc, USA), digitized at a 200 Hz sampling rate and stored for off-line analysis using Micro1401 MKII and Spike2 software (CED Co., UK). CSDs were counted for 1 hour at the parieto-occipital and frontal recording sites and the results were expressed as CSD frequency per hour. Propagation velocity between parieto-occipital and frontal recording sites was calculated for the first CSD.

### Statistical analysis

Group values were expressed as means ± standard error of means. To assess the effect of sexual hormones on L-KYN-induced CSD changes, two-way ANOVA [treatment (NaCl/L-KYN) x hormone (E2/E2Cont)] and [treatment (NaCl/L-KYN) x hormone (P4/P4Cont)] was used followed by Duncan's post-hoc tests. Analyses were implemented in Statistica (Version 9 for Windows) with p<0.05 as threshold for statistical significance.

## Results

### Influence of E2 on CSD frequency and its suppression by L-KYN

**Figure 1:**
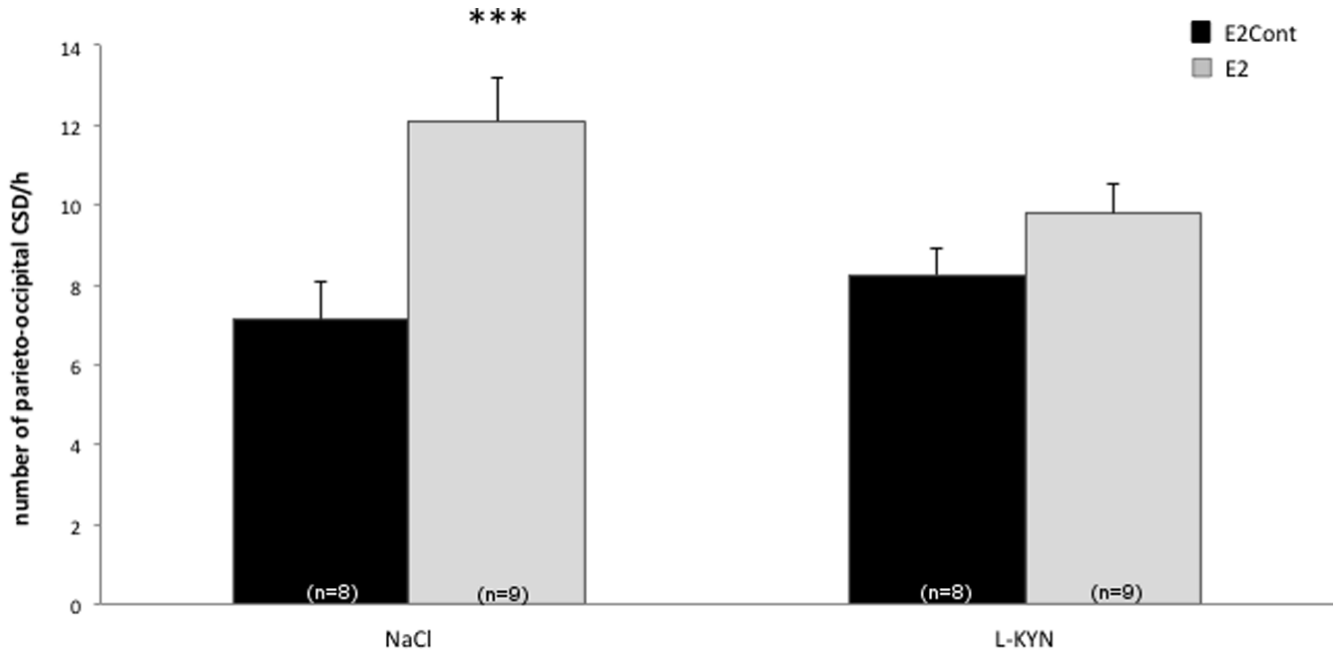
CSD frequency at the parieto-occipital site in rats treated with 17β-estradiol. Number of parieto-occipital CSD/hour in ovariectomized female rats treated during two weeks with cholesterol (E2Cont) or 17β-estradiol (E2) after i.p. injection of L-KYN or NaCl (***p<0.001 Duncan's test E2Cont NaCl vs E2 NaCl).

At the parieto-occipital level, ovariectomized females treated since 2 weeks with E2 had a higher CSD frequency than those treated with cholesterol. This effect appears for the global population (ANOVA hormone effects F(1,34) = 12.91, p = 0.001) and, in particular, for NaCl treated females (Duncan's test E2 vs E2Cont, p = 0.001) (Fig.1). The same result was obtained at the frontal recording site (Table 1). In NaCl groups, E2-treated females had a greater number of CSDs than those treated only with cholesterol (Duncan's test E2Cont vs E2, p = 0.005). The hormonal treatment had no influence on the propagation velocity of the first CSD (ANOVA hormone effects F(1,34) = 0.46, N.S.).

**Table 1:** CSD frequency at the frontal recording site and CSD propagation velocity in ovariectomized rats treated or not with estradiol.

		NaCl	L-KYN
CSD frequency at frontal recording site	E2Cont	5.25±0.31	8.25±0.68
	E2	12.11±1.09**	9.78±0.76
CSD propagation velocity (mm/min)	E2Cont	3.34±0.27	3.04±0.15
	E2	3.23±0.10	2.86±0,18

**p<0.01 Duncan's test E2Cont NaCl vs E2 NaCl; E2 L-KYN group n = 9; E2Cont L-KYN n = 8; E2 NaCl group n = 9; E2Cont NaCl n = 8.

L-KYN had no significant influence on CSD frequency in ovariectomized females neither at the parieto-occipital site (ANOVA treatment effect F(1,34) = 0.44, N.S.) (Fig.1), nor at the frontal site (ANOVA treatment effects F(1,34) = 1.30, N.S.) (Table 1). L-KYN numerically decreased parieto-occipital CSD frequency in ovariectomized E2-treated animals (mean: 9.7/h vs 12.11 for NaCl), but this effect only tended to be significant (Duncan's test NaCl vs L-KYN, p = 0.078). L-KYN administration had no significant effect on CSD propagation velocity (ANOVA treatment effects F(1,34) = 3.33, N.S.) (Table 1).

### Influence of P4 on CSD frequency and its suppression by L-KYN

**Figure 2:**
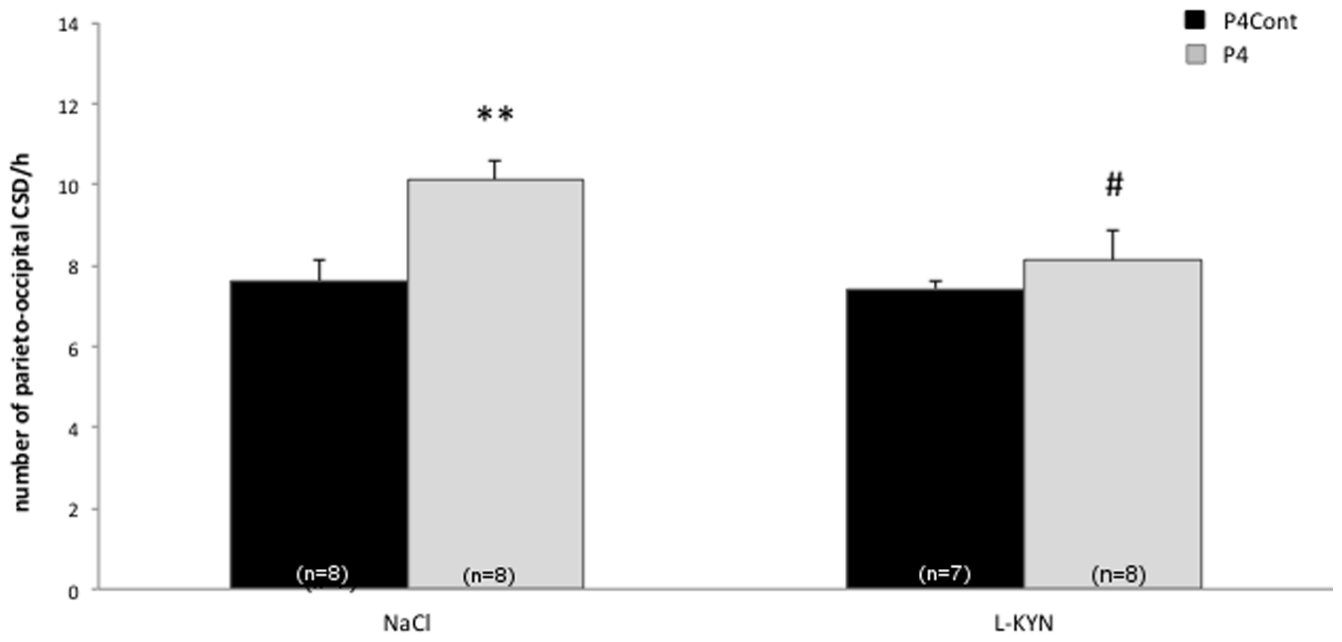
CSD frequency at the parieto-occipital site in rats treated with progesterone. Number of parieto-occipital CSD/hour in ovariectomized female rats treated during two weeks with progesterone (P4) or with empty capsules (P4Cont) after i.p. injection of L-KYN or NaCl (** p<0.01 Ducan's test P4 NaCl vs P4Cont NaCl; # p<0.05 P4 NaCl vs P4 L-KYN).

Implanted P4 capsules increased parieto-occipital CSD frequency after 2 weeks compared to implanted capsules left empty. This effect was significant for the global population of animals (ANOVA hormone effects F(1,31) = 8.98, p = 0.006) and in NaCl-treated females (Duncan's test P4 vs P4Cont, p = 0.004) (Fig. 2). L-KYN treatment significantly decreased parieto-occipital CSD frequency in P4 treated rats (Duncan's test NaCl vs L-KYN, p = 0.013) while it had no effect on CSD frequency in NaCl treated females.

By contrast, although progesterone numerically increased CSD frequency at the frontal recording site, there was no significant change after L-KYN treatment, neither of frontal CSD frequency nor of propagation velocity of the first CSD (frontal CSD frequency: ANOVA hormone effects F(1,31) = 0.11, N.S., ANOVA treatment effects F(1,31) = 1.42, N.S.; propagation velocity: ANOVA hormone effects F(1,31) = 0.06, N.S., ANOVA treatment effects F(1,31) = 0.42, N.S.) (Table 2).

**Table 2:** CSD frequency at the frontal recording site and CSD propagation velocity in ovariectomized rats treated or not with progesterone.

		NaCl	L-KYN
CSD frequency at frontal recording site	P4Cont	7.75±0.79	7.86±0.40
	P4	8.75±0.49	7.25±0.75
CSD propagation velocity (mm/min)	P4Cont	3.31±0.26	3.36±1.80
	P4	3.09±0.25	3.38±0.16

P4 L-KYN n = 8; P4Cont L-KYN n = 7; P4 NaCl n = 8; P4Cont NaCl n = 8.

## Discussion

Our results demonstrate for the first time that treatment with estradiol or progesterone in ovariectomized rats increases KCl-induced CSD frequency. Moreover, we found that the suppressive effect of systemic L-KYN administration on CSD previously reported in normally cycling females disappears after ovariectomy, but becomes apparent again after progesterone replacement treatment, but not after estrogen administration. We will discuss these two findings in sequence.

### Influence of female hormones on CSD frequency

The results obtained in ovariectomized rats with or without estrogen replacement therapy are in line with those reported in the knock-in mouse model of familial hemiplegic migraine type 1 [10]. In mutant R192Q and S218L mice the increased CSD susceptibility was decreased by ovariectomy and partially restored by estrogen treatment. In our previous study of the effects of L-KYN on CSD susceptibility, CSD frequency in NaCl-treated cycling females was on average 10.75 [12], which is clearly superior to the 7.13 and 7.63 frequency values found here in ovariectomized control animals in E2 and P4 groups respectively. This suggests that ovariectomy decreases CSD frequency in normal female rats as it does in the FHM1 mutant mice [10]. At variance with the latter is that in our study we found no effect of sex hormones on CSD propagation velocity.

Estrogens can modify susceptibility to CSD by several mechanisms, but mainly through their effect on glutamate neurotransmission that is known to subtend CSD generation. Estrogens affect neuronal plasticity during the estrous cycle by increasing the number of dendritic spines [20] and synaptic densities via an NMDA receptor dependent mechanism [21], [22]. They upregulate NMDA receptors and downregulate glutamate uptake by astrocytes [23], [24].

Contrary to estrogens, progesterone is classically thought to inhibit neuronal activity [25]. Progesterone can reduce cortical NMDA receptor binding density [26] and its metabolite, allopregnanolone, hyperpolarizes the neuronal membrane by increasing GABA mediated chloride conductance. These effects could decrease CSD susceptibility rather than increase it, as found in our study after P4 replacement therapy. Despite the lack of a clear-cut neurobiological explanation, at least one study found results that are similar to ours. Sachs et al. (2007) reported in a model of rat neocortical slices that addition of progesterone to the medium increased CSD amplitude and frequency, leaving propagation velocity unchanged. Progesterone had the same effect as estrogen and both hormones also promoted long-term potentiation [27]. In humans, there is only indirect evidence that progesterone may enhance neuronal excitability. Excitability of the motor cortex, as tested by transcranial magnetic stimulation, was increased in women with the premenstrual syndrome during the luteal phase when circulating progesterone levels were high [28].

In the present study, progesterone increases significantly CSD frequency at the parieto-occipital recording site, but only numerically at the frontal site, while estrogen has a significant enhancing effect at both recording sites. This difference may have various explanations. In general, the number of CSDs tends to be lower at the frontal than at the parieto-occipital recording site because a number of occipitally-generated CSD waves do not spread into the frontal cortex. Experimental interventions can differentially influence CSD generation and its postero-anterior propagation. For instance, we have found in a previous study [18] that chronic valproate treatment in rat decreased frontal but not parieto-occipital CSD frequency while lamotrigine had an inhibitory effect at both sites. Progesterone could have an opposite differential profile, increasing CSD generation close to the KCl application site, but inhibiting CSD propagation to the frontal recording site. The numerical, though non-significant, decrease of CSD propagation velocity after progesterone would favor this hypothesis. Alternatively, one may argue that the increase of frontal CSDs induced by estrogen treatment is amplified because of the low mean value of CSD frequency in the estrogen-control group (5.25) respective to the progesterone-control group (7.75). We do not think, however, that this is the explanation for the difference in frontal CSDs between estrogen and progesterone treatments. Also, the fact that the implanted silastic capsules were filled with cholesterol in E2Cont, but left empty in P4Cont, cannot explain this difference, since cholesterol would have influenced CSD propagation both in the E2Cont and E2 groups. Moreover, the presence of cholesterol had no influence on CSD occurrence, as evidenced by the similar CSD frequency at the parieto-occipital site in the estrogen and progesterone control groups (see figures 1 & 2).

CSD is thought to underlie aura symptoms [29] and the influence of hormones on MA has been attributed to an effect on CSD [25]. Our findings in rats are in line with data showing that in women persistent high gonadal steroid levels are deleterious for MA. During the first trimester of pregnancy, for instance, migraine symptoms may worsen in women suffering from MA and MA attacks may appear *de novo* during pregnancy [30]. Concordantly, combined oral contraceptives tend to worsen MA symptoms or to trigger the appearance of aura symptoms [5]. On the other hand, progesterone-only contraceptive pills had a beneficial effect on MA frequency and intensity in an observational prospective study [31]. The effect, however, took 6 months to appear and was observed only in women in whom MA onset was related to previous use of combined oral contraceptives. In another retrospective study of a mixed group of MO and MA patients [32] contraception with desogestrel decreased on average migraine frequency, intensity and use of any headache medication, except triptans, but out of 43 patients 7 had an increase in headache frequency. The weakness of both studies is the lack of a control group of women without hormonal treatment.

### Influence of female hormones on the suppression of CSD by L-KYN

In a previous study we have shown that the sole systemic administration of L-KYN at the same concentration as in the present work has a suppressive effect on CSD frequency in female rats, whereas in males we obtained such an effect only when L-KYN was combined to probenecid, an amino-acid transporter inhibitor increasing cerebral kynurenic acid concentrations [12]. The suppressive effect of L-KYN on CSD frequency is associated with an increase in cerebral levels of kynurenic acid. This effect varies with the phases of the estrous cycle. It is amplified during diestrus when progesterone levels are low and estrogen levels only start to raise [33], which indicates that sex hormones modulate the suppressive effect of L-KYN on CSD. In the present study, we have tested separately the effect of estradiol and progesterone on the L-KYN-induced suppression of CSDs in ovariectomized animals. While L-KYN is not different from NaCl in ovariectomized control rats, it reduces significantly the increase in parieto-occipital CSDs induced by progesterone replacement therapy and tends to do so for the one induced by estrogen. The latter effect does not reach the level of statistical significance probably due to the large variance of data. We assume that the administration of 300 mg/kg L-KYN alone does not increase brain KYNA sufficiently to inhibit CSD frequency in ovariectomized rats without hormonal replacement treatment.

There could be several explanations for these findings. First, estrogens are able to inhibit several enzymes involved in tryptophan metabolism, in particular kynurenine aminotransferase that synthesizes kynurenic acid from L-KYN [34], [35]. Although poorly studied, progesterone apparently does not inhibit kynurenic acid synthesis [35]. If the effect of female hormones on kynurenic acid metabolism would play a role, one would expect an enhanced inhibition of CSDs by L-KYN after ovariectomy, which is clearly not the case. Secondly, after ovariectomy there is no finely tuned cycling of hormone levels anymore. This is at variance with normally cycling females where the hormonal variations induce a number of genomic and non-genomic regulations in combination [36]. Moreover, in normal cycling females it is the interaction between estrogen and progesterone that shapes synaptic plasticity, glutamatergic neurotransmission and brain excitability (see above) [37]. The lack of a similar cycle-dependent interaction in ovariectomized rats without hormonal replacement treatment may contribute to the loss of significant CSD suppression by L-KYN, whereas with hormonal treatments the suppressive effect of L-KYN reappears or tends to reappear. Since L-KYN, i.e. kynurenic acid, modulates CSDs most likely by blocking NMDA receptors [13] and both estrogen and progesterone can modify these receptors [21]–[26], it seems plausible that the hormonal effects on L-KYN-induced CSD suppression are mediated via NMDA receptor changes. This hypothesis can be tested in a future study using NMDA receptor antagonists. Finally, as discussed above, in ovariectomized animals CSD frequency is markedly decreased compared to normal cycling rats whichever of the phase of the estrous cycle is considered [12]. The lack of a supplementary decrease of CSD frequency by L-KYN in ovariectomized rats without hormonal replacement might thus also be due to the fact that after ovariectomy a “floor” is reached beyond which no further effect can be expected from a compound acting on glutamate transmission. This would also account for the reappearance of some visible effect of L-KYN after the before-discussed enhancing effect on CSD frequency by estradiol or progesterone treatment.

## Conclusion

Hormone-dependent changes in cortical excitability contribute to the sexual dimorphism of migraine. We confirm here that ovariectomy in female rats decreases CSD frequency as well as the suppressive effect of L-KYN on CSD. We show in addition that both estrogen and progesterone replacement therapies increase CSD frequency and that progesterone restores the CSD inhibition by L-KYN. Taken together these results underscore the complex role of ovarian hormones in CSD susceptibility, and thus in migraine with aura, providing novel aspects to be taken into consideration for its management.
